# Synthesis, Characterization, In-Vitro and In-Vivo Evaluation of Ketorolac Tromethamine-Loaded Hydrogels of Glutamic Acid as Controlled Release Carrier

**DOI:** 10.3390/polym13203541

**Published:** 2021-10-14

**Authors:** Muhammad Suhail, Chuan-Ming Shih, Jia-Yu Liu, Wan-Chu Hsieh, Yu-Wen Lin, Muhammad Usman Minhas, Pao-Chu Wu

**Affiliations:** 1 School of Pharmacy, Kaohsiung Medical University, 100 Shih-Chuan 1st Road, Kaohsiung City 80708, Taiwan; suhailpharmacist26@gmail.com (M.S.); abcderic37@gmail.com (C.-M.S.); u109830006@kmu.edu.tw (J.-Y.L.); wanchuhsieh@gmail.com (W.-C.H.); u108530006@kmu.edu.tw (Y.-W.L.); 2 College of Pharmacy, University of Sargodha, Sargodha 40100, Pakistan; 3 Department of Medical Research, Kaohsiung Medical University Hospital, Kaohsiung 80708, Taiwan; 4 Drug Development and Value Creation Research Center, Kaohsiung Medical University, Kaohsiung 80708, Taiwan

**Keywords:** hydrogels, swelling study, drug release, in-vivo study

## Abstract

Glutamic acid-co-poly(acrylic acid) (GAcPAAc) hydrogels were prepared by the free radical polymerization technique using glutamic acid (GA) as a polymer, acrylic acid (AAc) as a monomer, ethylene glycol dimethylacrylate (EGDMA) as a cross-linker, and ammonium persulfate (APS) as an initiator. Increase in gel fraction was observed with the increasing concentration of glutamic acid, acrylic acid, and ethylene glycol dimethylacrylate. High percent porosity was indicated by developed hydrogels with the increase in the concentration of glutamic acid and acrylic acid, while a decrease was seen with the increasing concentration of EGDMA, respectively. Maximum swelling and drug release was exhibited at high pH 7.4 compared to low pH 1.2 by the newly synthesized hydrogels. Similarly, both swelling and drug release increased with the increasing concentration of glutamic acid and acrylic acid and decreased with the increase in ethylene glycol dimethylacrylate concentration. The drug release was considered as non-Fickian transport and partially controlled by viscoelastic relaxation of hydrogel. In-vivo study revealed that the AUC_0–∞_ of fabricated hydrogels significantly increased compared to the drug solution and commercial product Keten. Hence, the results indicated that the developed hydrogels could be used as a suitable carrier for controlled drug delivery.

## 1. Introduction

Non-steroidal anti-inflammatory drugs (NSAIDs) are the most commonly used drugs by patients over 60 years of age, especially those who are on NSAID medication [1]. NSAIDs have minor side effects and greater analgesic potency compared to opioids; hence, they are commonly used for the management of acute pain, such as headaches, stomach aches, the flu, etc. Ketorolac tromethamine (KETM) is a NSAID used to relieve severe pain with low anti-inflammatory and high analgesic activity [2,3]. The available dosage forms of KETM on the market are tablets, injections, drops, or ophthalmic solutions with dosage regimens of 10 mg, 15–30 mg/mL, and 0.5%, respectively. Similarly, in the UK, the available dosage forms of KETM on the market are tablets, injections, ophthalmic drops (0.5%), and solutions for injection (30 mg/mL). The half-life of KETM is within the range of 2.5–4 h. Administering KETM doses is conducted anywhere from one to multiple times in a day due to its short half-life, in order to maintain the therapeutic levels for an extended period of time, which produce certain complications, such as gastrointestinal ulceration, gastrointestinal bleeding, perforation and peptic ulceration, and acute renal failure [4]. Patient compliance is also reduced due to frequent administration of KETM. Hence, a suitable drug carrier system is needed to prolong the release of KETM as a controlled drug delivery system to overcome the complications concerned with the frequent administration of KETM [5]. Therefore, different advanced and controlled drug delivery strategies, such as dendrimers, nanocapsules, liposomes, nanofibers, hydrogels, and hydrogel nanoparticles are used for the delivery of therapeutic agents to the target sites in order to overcome the detrimental side effects. Among them, hydrogels are considered the most effective drug carrier systems due to their unique characteristics, such as high porosity, flexibility, biocompatibility, biodegradability, and high water intake content [6,7].

Hydrogels are three-dimensional structures, which are prepared by the crosslinking of hydrophilic polymers, and swelled in water solution without losing their structural consistency. The desirable water absorption or retention of hydrogels depends on their porosity, which provides greater surface area and, thus, a greater amount of water is absorbed [8,9]. Stimuli-responsive hydrogels or smart hydrogels have gained much interest, particularly in the biomedical field, due to their stimulus sensitive nature compared to conventional hydrogels. Due to the presence of stimuli responsive or smart polymers [10], stimuli-responsive polymer based hydrogels can respond to external stimuli, i.e., pH, temperature, and ionic strength and play an important role in gene delivery, drug delivery, and tissue regeneration. pH-sensitive hydrogels have been widely studied among stimuli responsive hydrogels because of their unique properties as they release the drug to the target site in a controlled way by regulating their swelling behavior at that particular site [11].

Glutamic acid (GA) is water soluble, biodegradable, edible, and nontoxic in regard to individuals and the environment. Hence, GA and its derivatives are considered of a great interest in both pharmaceutical and industrial fields, such as medicine, cosmetics, food, and water treatment [12]. GA based polymers have been widely investigated, especially for biomedical and pharmaceutical purposes for many years. The structure and features of the final polymer are affected directly by the type of linkage between the GA monomers and by the position of the amino acid group either within the main chain or pendant to it. Biodegradability and stability of polymers depend on the structural arrangement of the amino acid units. Polymers will be biodegradable if the amino acid units are exhibited on their backbone and will be stable, hydrolytically highly, if the amino acid units are attached as a side chain [13]. Acrylic acid (AAc) is a pH sensitive polymer and plays an important role in controlled drug delivery systems due to its ionic nature. AAc has a COOH functional group, which protonates at a lower pH and deprotonates at a higher pH. Due to deprotonation, AAc swells highly at upper pH values and lower at lower pH values due to protonation. The bio-compatibility of AAc is because of COOH functional groups. Due to good bioadhesive nature, AAc is used widely for the enhancement of the retention time of formulation [14].

Here, we report the development of GAcPAAc hydrogels used for the controlled delivery of KETM. Nine formulations with various concentrations of GA, AAc, and EGDMA were prepared by free radical polymerization. Different characterizations, such as FTIR, TGA, DSC, PXRD, and SEM were carried out to assess and evaluate the different parameters of the fabricated hydrogels. Similarly, studies such as sol–gel analysis, dynamic swelling, drug loading, in-vitro drug release, and in-vivo experiments were performed to understand the compatibility and sustainability of the GAcPAAc hydrogels.

## 2. Materials and Methods

### 2.1. Materials

Glutamic acid was procured from Across Organic, Janssen Pharmaceuticalaan, Belgium. Ketorolac tromethamine (KETM) was obtained from Symed Labs Limited (Telangana, India). Acrylic acid (AAc) was purchased from Acros (Carlsbad, CA, USA). Ketoprofen was obtained from Sigma-Aldrich (St. Louis, MO, USA). Similarly, ammonium persulfate (APS) and ethylene glycol dimethacrylate (EGDMA) were obtained from Showa (Tokyo, Japan) and Alfa-Aesar (Tewksbury, MA, USA). Commercial product: keten capsule was obtained from Everest Pharm. Industrial Co., LTD. (Taiwan).

### 2.2. Preparation of GAcPAAc Hydrogels

**Figure 1:**
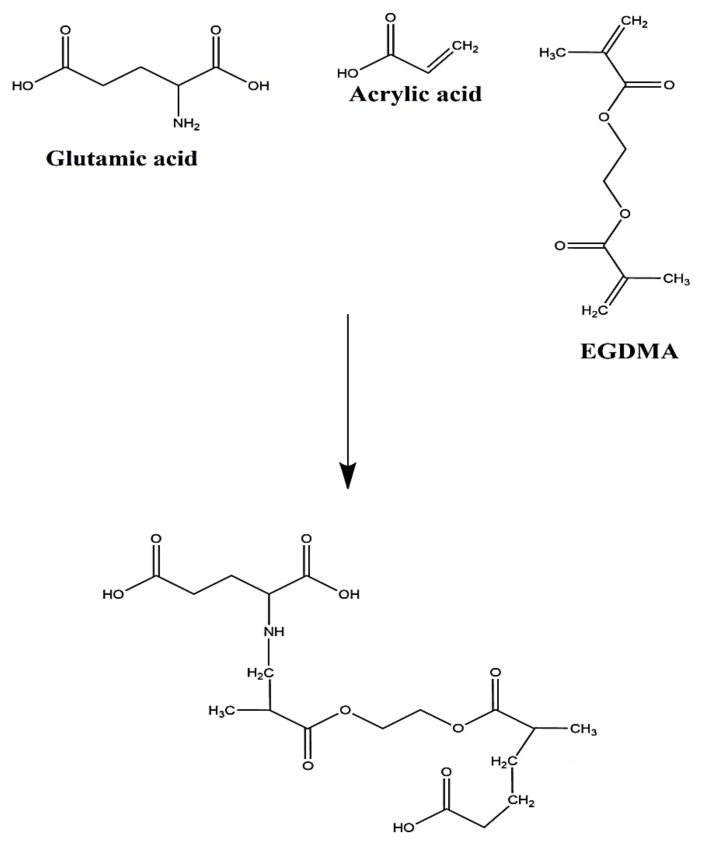
Proposed chemical structure of GAcPAAc hydrogels.

Various formulations of glutamic acid-co-poly(acrylic acid) (GAcPAAc) hydrogels were synthesized by the free radical polymerization technique with different concentration of polymer GA, monomer AAc, and cross-linker EGDMA, as shown in Table 1. Initiator APS was used in a small fixed quantity throughout all formulations. Fixed weighed quantity of GA was taken and dissolved in a specific amount of deionized distilled water. Similarly, a fixed quantity of APS was dissolved in distilled water. Accurate amounts of AAc and EGDMA were taken separately. Initially the APS solution was added into the GA solution, followed by the dropwise addition of AAc. The mixture was stirred for 25 min on a magnetic stirrer (Corning PC-420D). Finally, EGDMA was added into the mixture of polymer, monomer, and initiator. The mixture was stirred until a transparent solution was formed. Then, the transparent solution was purged by nitrogen gas (Jing Shang, Kaohsiung, Taiwan) in a such a way that the nozzle (through which nitrogen gas was released) was kept slightly away from the surface of the solution in order to remove any dissolved oxygen completely if present in the solution. The transparent solution was transferred into the glass molds and kept in a water bath at 55 °C initially for 2 h, and then the temperature increased to 65 °C for the next 22 h. After 22 h, the gel was formed, cut into 8 mm discs equally, and then washed by the mixture of water and ethanol (50% *v*/*v*) to remove any impurity attached on the surface of the gel. All the discs were placed in a vacuum oven at 40 °C for one week after placing them at room temperature for 24 h initially. The dried discs were assessed for further studies. The proposed chemical structure of GAcPAAc hydrogels is given in Figure 1.

### 2.3. Sol–Gel Analysis

Sol–gel analysis was carried out for all developed formulations to know the soluble un-crosslinked and insoluble crosslinked portions of the fabricated hydrogels. Gel is the insoluble while sol is the soluble fraction of the hydrogels. Hence, the Soxhlet extraction technique was conducted for the sol–gel analysis. The accurate amount of hydrogel disc was taken and placed in a round bottom flask containing a specific volume of deionized distilled water. A condenser was connected to the round bottom flask. The extraction process continued for 13 h at 85 °C. Afterward, the hydrogel disc was extracted, placed in the vacuum oven until completely dehydrated, and weighed again [15]. Sol–gel analysis was determined by the given equations:

(1) $$\mathrm{Sol}\;\mathrm{fraction}\;\%=\frac{{\;Z}_{1}-{\;Z}_{2}}{{\;Z}_{2}}\times 100$$

(2) Gel fraction=100−Sol fraction

Z_1_ indicates the initial weight of hydrogels (before extraction), and Z_2_ shows the final weight of dried hydrogels (after extraction).

### 2.4. Fourier Transform Infrared Spectroscopy (FTIR) Analysis

FTIR spectra for GA, AAc, unloaded GAcPAAc hydrogels, KETM, and drug loaded GAcPAAc hydrogels were carried out to determine the structural arrangement of the components used in the development of hydrogels. All samples were crushed and then evaluated by using NICOLET 380 FTIR (Thermo Fisher Scientific, Ishioka, Japan) within the spectra range of 4000–500 cm^−1^ [16].

### 2.5. Thermal Analysis

Thermal analysis was performed for GA and GAcPAAc hydrogels by thermogravimetric analysis (TGA) (PerkinElmer Simultaneous Thermal Analyzer STA 8000) and differential scanning calorimetry (DSC) (PerkinElmer DSC 4000) to evaluate and compare the thermal stabilities of GA and fabricated hydrogels. Hence, the hydrogel discs were crushed and the desired sizes of particles were obtained after passing through mesh 40. Thus, 0.3–7 mg of particles were taken for TGA analysis and placed in an open pan connected to a microbalance. The samples were heated within temperature range of 40–600 °C under dry nitrogen flow. Similarly, 0.3–5 mg of particles was taken in an aluminum pan for DSC analysis of GA and GAcPAAc hydrogels. The samples were heated at a temperature range of 50–400 °C while the nitrogen flow and heating rate was kept at 20 mL/min and 20 °C/min throughout sample analysis [17].

### 2.6. Porosity Study

A solvent replacement technique was used for evaluation and analysis of porosity of all formulations of GAcPAAc hydrogels. Accurate weight of hydrogel discs (Q_1_) of all formulations were immersed in absolute ethanol (purity > 99.9%) for 4 days. After 4 days, hydrogel discs were taken out, blotted with filter paper to eliminate excess of solvent, and weighed again (Q_2_). Similarly, thickness and diameter of the discs were measured. The given equation was used for the determination of porosity [18].

(3) $$\mathrm{Porosity}\;\mathrm{percentage}\;(\%)=\;\frac{Q_{2}-Q_{1}}{\rho V}\times 100$$

*ρ* indicates the density of absolute ethanol, while V shows the volume of hydrogel after swelling.

### 2.7. Dynamic Swelling Study

A swelling study was performed for all formulations of the fabricated hydrogels to know the swelling degree at two different pH values i.e., pH 1.2 and 7.4, at 37 °C. Therefore, a weighed amount of a hydrogels disc was placed in the respective pH medium. After a regular interval of time, the hydrogels disc was taken out, blotted with filter paper to remove excess of water, and weighed again on weighing balance. This process was continued until an equilibrium weight was obtained [19]. This experiment was performed in triplicate. The given equations were used to calculate the dynamic swelling and equilibrium swelling, respectively:

(4) $$\;q=\frac{{\;D}_{2}}{{\;D}_{1}}$$

where q represents dynamic swelling, D_1_ is the initial weight of the dried hydrogel discs before swelling, and D_2_ is the final weight of the swelled hydrogel discs at time t.

(5) $$\mathrm{SR}\%=\frac{{\;P}_{1}-{\;P}_{2}}{P_{2}}\times 100$$

where P_1_ is the weight of the swollen hydrogel discs at specific times, while P_2_ is the weight of the dry hydrogel discs before swelling.

### 2.8. Polymer Volume Fraction Study

Polymer volume fraction study was conducted for all formulations of GAcPAAc hydrogels to determine the fraction of the polymer in the swelled states at both pH 1.2 and 7.4, respectively. It is denoted by V2. Equilibrium volume swelling (Veq) data were employed for the determination of polymer volume fraction [20]. Therefore, the given equation was used;

(6) $$V2,s=\frac{1}{\mathrm{Veq}}$$

### 2.9. Scanning Electron Microscopy (SEM)

Surface morphology of GAcPAAc hydrogels was determined by SEM (JSM-5300 model (JEOL, Tokyo, Japan). Surface morphology of developed hydrogels was scanned by various magnifications [21].

### 2.10. Powder X-ray Diffractometry (PXRD) Analysis

PXRD (XRD-6000 Shimadzu, Tokyo, Japan) analysis was carried out for GA and GAcPAAc hydrogels to evaluate the crystallinity of pure GA and developed hydrogels at room temperature. Samples were placed in a plastic sample holder and their surfaces were leveled by a glass slide. Theta (θ) was kept within the range of 10^°^–60^°^ with a rate of 2^°^ 2θ/min at 25 °C throughout the samples analysis [22].

### 2.11. Drug Loading

Loading of KETM was carried out for all formulations of GAcPAAc hydrogels by absorption and diffusion method. Therefore, weighed hydrogel discs were placed in 1% drug solution of phosphate buffer pH 7.4 for four days. After equilibrium swelling and loading, hydrogel discs were removed and washed by distilled water to remove the excess drug attached with the surface of the hydrogel discs. Afterward, the hydrogel discs were placed in a vacuum oven for dehydration at 40 °C [23].

Quantification of the loaded drug by fabricated hydrogels was conducted by two methods: 1) extraction method, and ii) weight method. In the extraction method, accurate weight-loaded hydrogel discs were immersed in 25 ml of phosphate buffer of pH 7.4. After predetermined intervals, phosphate buffer solution was replaced by fresh buffer solution of the same pH (7.4) and samples were collected. This process continued until the entire drug was eliminated from the loaded hydrogel discs. The collected samples were then analyzed on UV–vis-spectrophotometer (U-5100, 3J2-0014, Tokyo, Japan) at λmax 280 nm in triplicate [19].

In the weight method, the weight difference between the loaded and unloaded hydrogel discs was determined; hence, the weight of the unloaded dried hydrogel discs was subtracted from the weight of the loaded dried hydrogel discs [24] as indicated in the given equation:

Amount of drug loaded = M_L_ − M_UL_ (7)

where M_L_ = weight of loaded disc of hydrogels, and M_UL_ = weight of unloaded disc of hydrogels.

### 2.12. In-Vitro Drug Release Study

In-vitro drug release studies were performed for commercial product Keten and GAcPAAc hydrogels at both acidic and basic media i.e., pH 1.2, and 7.4 to analyze the pH sensitive nature of the fabricated hydrogels. Keten and loaded hydrogel discs of all formulations with a height and diameter of 8 and 10 mm were immersed in buffer solution of 900 mL of both pH 1.2 and 7.4 using USP dissolution apparatus II (Sr8plus Dissolution Test Station, Hanson Research, Chatsworth, CA, USA) at 37 ± 0.5 °C. A sample of 5 mL was taken after a specific interval of time, and fresh medium of the same quantity was added back to keep the sink condition constant. UV–vis-spectrophotometer (U-5100, 3J2-0014, Tokyo, Japan) was used for the analysis of drug contents in the collected samples at a wavelength (λmax) of 280 nm in triplicate [25].

### 2.13. Kinetic Modeling

The release data fit in different kinetic models, such as zero order, first order, Higuchi and Korsmeyer–Peppas models, to deduce the release mechanism of KETM from GAcPAAc hydrogels [26].

### 2.14. In-Vivo Study

Livestock Research Institute, council of Agriculture Executive Yuan, Taiwan provided the female/male New Zealand rabbits weighed of 2.5–3.0 kg. This study was approved (no. 109008) by the Committee on Animal Use of Kaohsiung Medical University. The experiment was carried out according to the guidelines of Kaohsiung Medical University for the welfare and ethics of the experimental animals. A good healthy environment was provided to the rabbits. Nine rabbits were chosen for the experiment and divided into three groups, i.e., group I (drug solution group), group II (commercial product, Keten), and group III (test hydrogel group). Standard food was given to all of the rabbits before the start of the study. Before the dose administration, food was stopped and made fast for 12 h over-night. The rabbits were stained and placed in the boxes. By the help of a feeding tube, drug solution (10 mg/kg) was given to group I, followed by rinsing with 1 mL of water. Similarly, commercial product keten (10 mg/kg) and loaded GAcPAAc (25.57 mg/kg) were given orally to group II and group III, respectively. Moreover, 0.6 mL blood samples were taken from the ear vein of all three groups at specific time intervals and stored in a heparinized polypropylene tube. Then, centrifugation for samples was carried out for 10 min at 4000 rpm to obtain plasma [27]. KETM contents in plasma were determined by high performance liquid chromatography (HPLC, HITACHI). Column (LiChrospher^®^100 RP-18 endcapped 250 × 4 mm, 5 μm (Merck, Darmstadt, Germany) was used for the detection of KETM (RT: KETM = 5.1 min, internal standard of ketoprofen = 7.7 min (400 μg/mL methanol)). The mixture of phosphoric acid (pH 3.0)/acetonitrile (55:45, *v*/*v*), respectively, was used as a mobile phase at a flow rate of 1.2 mL/min. A mixture ( plasma = 100 uL and internal standard = 100 uL) was taken, and kept on vortex mixing for 1 min. Centrifugation was carried out at 8000 rpm for 5 min at 25 °C. KETM was detected at λ max 313 nm [28]. Pharmacokinetic parameters: the maximum concentration (Cmax), time to achieve Cmax (Tmax), elimination rate constant (K), elimination half-life (t_1/2_), and area under the curve after extrapolation from time x to infinit (AUC_0–∞_) were taken directly from the concentration course or calculated by Excell software.

### 2.15. Statistical Analysis

Statistical analysis was performed by using a computer program, SPSS Statistic software 22.0 (IBM Corp, Armonk, NY, USA). By using Student’s t-Test, differences between tests were tested and considered statistically significant because the *p*-value was <0.05.

## 3. Results

### 3.1. Sol-Gel Analysis

Sol-gel analysis is performed for all formulations of GAcPAAc hydrogels as indicated in Table 1. Hydrogels contents influence the sol and gel fractions of the fabricated hydrogels. Sol fraction is decreased with the increase in the concentration of GA, AAc, and EGDMA because there is an inverse relationship between the sol fraction and gel fraction [29]. An increase of one fraction leads to a decrease in another fraction. Gel fraction increases with the increase in the concentration of GA (Table 1). The main reason is the increase in availability of free radicals for AAc during polymerization reaction. The higher the free radicals, the faster the polymerization reaction and, hence, the greater the gel fraction will be (and vice versa). Similarly, an increase in gel fraction is observed as the concentration of AAc is increased (Table 1). High numbers of functional groups (carboxylic groups) of AAc are generated, which leads to higher polymerization of AAc with the GA, and as a result gel fraction is enhanced. Similar to GA and AAc, an increase in concentration of EGDMA leads to maximum crosslinking, and as a result, GA and AA are crosslinked rapidly, and gel fraction increases (Table 1). The compatibility of hydrogels depends on the concentration of the cross-linker used. The greater the cross-linker, the higher the polymerization reaction, and the greater the gel fraction (and vice versa) [30,31]. Khalid and his co-workers prepared CS-co-p(AMPS) hydrogels and reported an increase in the gel fraction with the increase in the concentration of polymer, monomer, and cross-linker, while a decrease in sol–fraction was observed and vice versa [32].

### 3.2. Fourier Transform Infrared Spectroscopy (FTIR) Analysis

**Figure 2:**
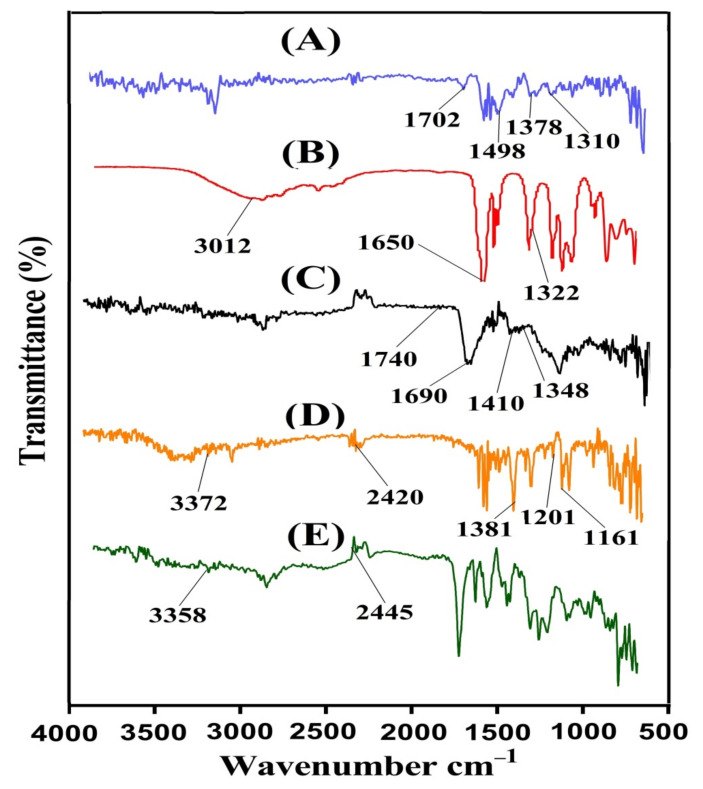
FTIR spectra of (**A**) GA, (**B**) AAc, (**C**) unloaded GAcPAAc hydrogels, (**D**) KETM, and (**E**) loaded GAcPAAc hydrogels.

FTIR analysis is carried out for GA, AAc, unloaded GAcPAAc hydrogels, KETM, and loaded GAcPAAc hydrogels as shown in Figure 2A–E. FTIR spectra of GA (Figure 2A) indicate stretching vibration of the carboxylic acid group by a peak at 1398 cm^−1^, while the three peaks assigned at 1702, 1498, and 1310 cm^−1^ are due to amide I, amide II, and amide III, respectively [33,34]. Similarly, FTIR spectrum of AAc (Figure 2B) indicates stretching vibration of –C–C and –CH_2_ by peaks at 1650 and 3012 cm^−1^, while –C=O stretching vibration is indicated by a peak at 1322 cm^−1^ [35]. Due to the electrostatic interaction between the GA and AAc, a modification in their peaks is observed in unloaded GAcPAAc hydrogels. The peaks of GA at 1398 and 1702 cm^−1^ are changed to 1410 and 1740 cm^−1^ peaks of unloaded GAcPAAc hydrogels (Figure 2C). Similarly, a few peaks of AAc are also changed from 1322 and 1650 cm^−1^ to 1348 and 1690 cm^−1^ peaks of unloaded GAcPAAc hydrogels. Some new peaks are formed; few disappeared while some altered. The alteration, disappearance, and formation of the new peaks depict the grafting of AAc over the GA backbone and, thus, indicate the development of GAcPAAc hydrogels. The prominent peaks of KETM (Figure 2D) are assigned by FTIR spectra at 3372, 1381, 1161, and 1201 cm^−1^, representing N-H and NH2, –C–N, C = O, and –OH stretching vibration. Likewise, C–H bending is observed by peaks at 2420 and 803 cm^−1^, respectively [36,37,38]. Due to the loading of KETM by the fabricated hydrogels, a slight modification is observed in the distinctive peaks of KETM as indicated in FTIR spectra of drug loaded GAcPAAc hydrogels (Figure 2E). The peaks of KETM at 3372 and 2420 cm^−1^ are altered slightly to 3358 and 2445 cm^−1^ peaks of the drug loaded GAcPAAc hydrogels. These all indicate the successful encapsulation of KETM by the fabricated hydrogels and, thus, no interaction is detected between the KETM and hydrogel contents [39].

### 3.3. Thermal Analysis

**Figure 3:**
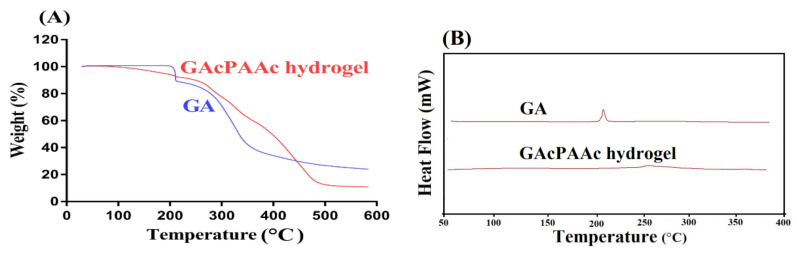
(**A**) TGA and (**B**) DSC of GA and GAcPAAc hydrogel.

Thermogravimetric analysis (TGA) is performed for the purpose to know the thermal stability of GA and GAcPAAc hydrogels, as shown in Figure 3A. TGA of GA indicates no loss in weight up to 198 °C. Further increase in temperature leads to a decrease in weight of GA and a 22% loss in weight is observed as the temperature approaches 208 °C, which is the melting point T_M_ of GA. As temperature reaches 350 °C, weight loss of 58% is detected. After that, degradation of GA is started until complete pyrolysis [40]. TGA of GAcPAAc hydrogels reveal weight loss of 8% as the temperature approaches 210 °C. A further loss of 54% in weight is detected within a temperature range of 210–380 °C. Finally, degradation of GAcPAAc hydrogels starts at 480 °C until its complete degradation. Conclusively, the TGA thermogram of pure GA indicates that degradation half-life of GA, i.e., (t1/2 = 350 °C) is not greater than the degradation half-life of GAcPAAc hydrogels, i.e., (t1/2 = 480 °C), respectively. Therefore, we can conclude from the TGA thermogram of both GA and GAcPAAc hydrogels that thermal stability of pure GA is less than the thermal stability of the fabricated hydrogels. The possible reason for the higher thermal stability of GAcPAAc could be the crosslinking and grafting of hydrogel contents during the polymerization process. Barkat and coworkers prepared chondroitin sulfate based hydrogels and reported higher thermal stability for the designed hydrogels compared to pure polymer [32].

Differential scanning calorimetry (DSC) analysis is carried out for unreacted GA and GAcPAAc hydrogels as indicated in Figure 3B. GA exhibits an endothermic peak at 198 °C, followed by a glass transition temperature (T_g_), whereas an exothermic peak at 215 °C, which is followed by a melting (T_m_) or decomposition temperature (T_d_), respectively [41]. Similarly, DSC of GAcPAAc hydrogels exhibits an exothermic peak at 255 °C, which is the exothermic peak of GA, shifted from 215 °C to 255 °C due to the electrostatic interaction with others hydrogel contents. The endothermic peak of GA at 198 °C shifted to 210 °C in GAcPAAc hydrogels. Onward temperature leads to degradation of GAcPAAc hydrogels that indicate greater stability of developed polymeric hydrogels compared to unreacted GA [42].

### 3.4. Porosity Study

The swelling, loading, and drug release of hydrogel depends on its porosity. The greater the pore size, the higher the swelling, and as a result, the greater the drug loading and release. Porosity of fabricated hydrogels is increased with the increase in GA and AAc concentration (Figure 6A). The increase in porosity is due to the high viscosity of the reaction mixture that prevents the escape of bubbles from the reaction mixture. This all leads to generation of interconnected channels and, thus, increase in porosity is observed. Contrary to GA and AAc, a drop is seen in porosity with the enhancement of EGDMA concentration (Figure 6A) due to the formation of strong tight junctions and crosslinked bulk density that affect the drug flexibility [43].

### 3.5. Dynamic Swelling Study

**Figure 4:**
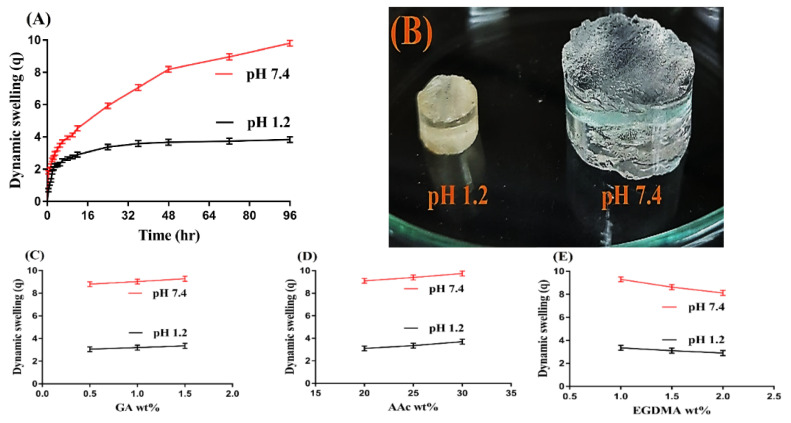
(**A**) Effect of pH on dynamic swelling, (**B**) swelled hydrogel at pH 1.2 and 7.4; effects of (**C**) GA, (**D**) AAc, and (**E**) EGDMA on dynamic swelling of GAcPAAc hydrogels.

The dynamic swelling study was performed for all formulations of GAcPAAc hydrogels to analyze the pH sensitive nature of the fabricated hydrogels at two different pH values, i.e., pH 1.2 and 7.4, respectively. pH influences the swelling of hydrogels highly as shown in Figure 4A. Low swelling is detected almost at pH 1.2 due to the protonation of functional groups of both GA and AAc. Both polymer and monomer contain carboxylic groups (COOH groups), which protonate at low pH 1.2 and form conjugate with the counter ions, which leads to a decrease in free charge density of the same groups. Strong hydrogen bonding is produced and the ionization process is decreased. These all lead to low swelling at pH 1.2. Contrary to pH 1.2, maximum swelling is exhibited by the fabricated hydrogels at high pH 7.4. As the pH of the medium is changed from lower to upper pH values, deprotonation of carboxylic groups of GA and AA occurs, which leads to generation of high charge density of the same group, and as a result, strong electrostatic repulsive forces are produced. These repulsive forces repeal the same charge particles and, thus, increase in swelling is perceived at high pH 7.4 [44,45].

The concentration of the hydrogel contents also influence the dynamic swelling at both pH 1.2 and 7.4, respectively, as shown in Figure 4C–E. An increase in the swelling index is seen with the increase in the composition of GA and AAc (Figure 4C,D). The possible reason is the generation of a high number of COOH groups with the increase in GA and AAc concentration, which produce high charge density and strong electrostatic repulsive forces. The same charge particles will repel each other and, hence, an increase in swelling will be observed [24,46,47]. Unlikely to GA and AAc, a drop in swelling is seen with the increase in EGDMA concentration (Figure 4E). The bulk density of the hydrogels increases with the increase in the concentration of EGDMA, due to which the porosity of the hydrogels decreases. Hence, penetration of water into the hydrogels network is decreased, and as a result, a reduction in swelling is exhibited with the increase in the concentration of EGDMA and vice versa [48,49,50,51].

### 3.6. Polymer Volume Fraction Study

Polymer volume fraction study is carried out for all formulations of fabricated hydrogels at both pH 1.2 and 7.4 as shown in Table 1, and greater polymer volume fraction is observed at pH 1.2 compared to pH 7.4. The composition of hydrogels contents greatly affects the polymer volume fraction. As the composition of GA and AAc is increased, the polymer volume fraction is decreased. Unlikely GA and AAc, an increase in polymer fraction is observed with the increase in composition of EGDMA. The reason could be interlinked with the swelling behavior of the fabricated hydrogels. The high values of polymer volume fraction at pH 1.2 and low at 7.4 are the indication of significant swelling and pronounced expansion ability of the developed hydrogels [20].

### 3.7. Scanning Electron Microscopy

**Figure 5:**
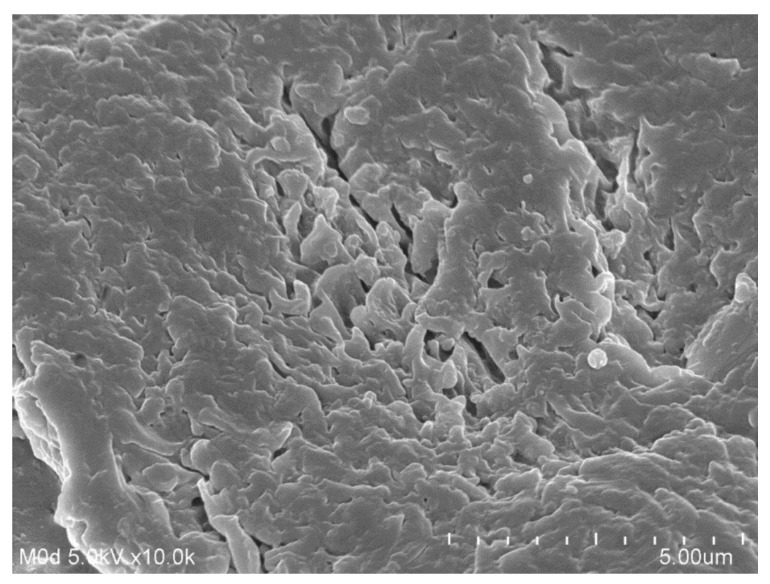
Scanning electron microscopy of GAcPAAc hydrogels.

Scanning electron microscopy (SEM) is carried out for fabricated hydrogels to analyze its surface morphology. A smooth and porous surface is indicated by the developed hydrogels as shown in Figure 5. The porous surface formed may be due to the evaporation of water occurring due to heat reaction. These pores may be supposed to act as a permeation channels to allow water molecules to penetrate inside the hydrogel network, and also provide site of active interaction for hydrophilic groups of the polymeric network and external stimuli [52].

### 3.8. Powder X-ray Diffractometry (PXRD) Analysis

**Figure 6:**
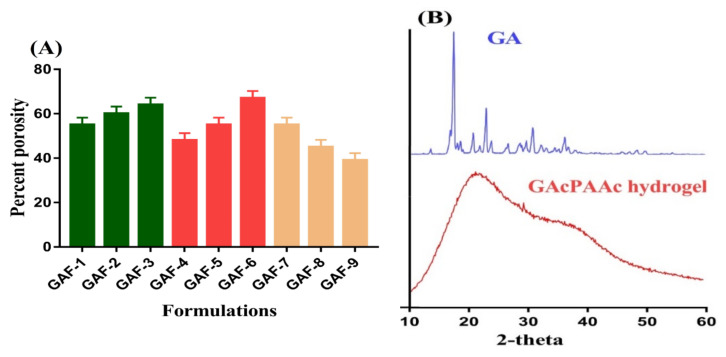
(**A**) Percent porosity and (**B**) PXRD of GA and GAcPAAc hydrogel.

PXRD is performed for the purpose of evaluating the crystallinity of the polymeric carrier system and the components used in its development. Hence, in the present study, PXRD is conducted for GA and GAcPAAc hydrogels to analyze their crystallinity as shown in Figure 6B. The crystalline peaks of GA are indicated at 2θ = 18.39°, 22.14°, 24.89°, and 32.04° by PXRD analysis, which reveal the crystallinity of GA. Similarly, PXRD analysis of GAcPAAc hydrogels indicates less crystalline nature due to the crosslinking of their components. The crystalline peaks of the GA disappear in the fabricated hydrogels due to the polymerization of GA with AAc. Lee et al. developed amphiphilic poly(l-lactide)-grafted chondroitin sulfate copolymer hydrogel and reported a reduction in the crystallinity of individual ingredients used in the development of the polymerized network of hydrogels [53].

### 3.9. Drug Loading

Drug loading is performed for all formulations of the developed hydrogels in order to analyze and evaluate the amount of drug encapsulated by the hydrogel content as indicated in Table 2. Swelling plays an important role in drug loading as the maximum amount of drug is loaded by the hydrogels if the swelling of the hydrogels is high. Therefore, for drug loading, such solvent is used within drug solubility and swelling of hydrogels is high. Drug loading is increased with the increase in the concentration of GA and AAc, as shown in Table 2. The possible reason is the greater swelling index of both GA and AAc, which leads to greater drug loading. Similar to swelling, a decrease in drug loading is observed due to high crosslinked density and less porosity as the concentration of EGDMA is increased (Table 2) [54].

### 3.10. In-Vitro Drug Release Study

**Figure 7:**
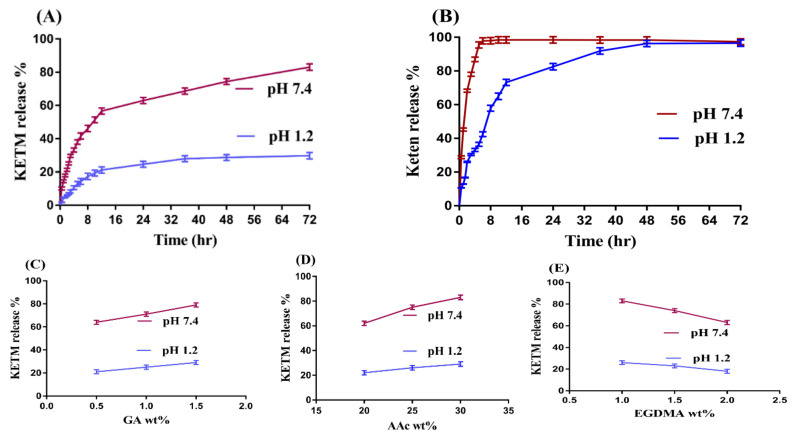
Effects of (**A**) pH, (**B**) commercial product Keten, (**C**) GA, (**D**) AAc, and (**E**) EGDMA on KETM percent release from GAcPAAc hydrogels.

An in-vitro drug release study is performed to know the release of drug from the keten capsule and the pH dependent release of drug from all formulations of the fabricated hydrogels at both acidic and basic medium as shown in Figure 7A–E. pH affects the percent of the drug released from the developed hydrogels as a low release of drug is depicted at acidic pH 1.2 compared to basic pH 7.4 (Figure 7A). The functional groups (COOH groups) of GA and AAc protonate at low pH, due to which charge density of the same groups is decreased because conjugates are formed due to strong hydrogen bonding and, thus, low swelling and the percent of the drug released is observed. On the other side, a high percent of the drug released is seen at high basic pH 7.4 due to the deprotonation of functional groups of the GA and AAc. Strong electrostatic repulsive forces are produced among the same charge groups due to high charge density as the pH of the medium is enhanced from pH 1.2 to pH 7.4, which repeal each other and lead to higher swelling and the percent of the drug released. Similarly, as shown in Figure 7B, a drug release study is carried out for the keten capsule at both pH 1.2 and 7.4, respectively. A high amount of drug is released at basic pH 7.4 as compared to acidic pH 1.2. A high percent of the drug released of 95% is observed within the initial 5 h at pH 7.4, and continues to 72 h. While at pH 1.2, drug release of 97% is detected at 72 h [55].

Percent of the drug released is also influenced by the different concentrations of GA, AAc, and EGDMA at both pH 1.2 and 7.4, respectively, as indicated in Figure 7C–E. The percent of the drug released increases as the concentration of GA and AAc increases (Figure 7C,D), because a greater quantity of carboxylic groups (COOH) is produced, leading to higher swelling and a percent of the drug released [56,57,58]. Similar to swelling, a reduction in the percent of the drug released is seen as the concentration of the EGDMA is increased (Figure 7E) due to higher crosslinking and bulk density and less porosity [59]. Rokhade and coworkers prepared ketorolac tromethamine loaded microspheres of gelatin and sodium carboxymethyl cellulose and reported a percent of the drug released for 10 h [60]. Similarly, Dasankoppa and coworkers prepared a controlled porosity osmotic pump for oral delivery of ketorolac and demonstrated the percent of the drug released for 12 h [61]. Comparing the percent of the drug released of the current study with previously published reported studies indicates that the developed network of GAcPAAc hydrogels prolongs the release of ketorolac tromethamine significantly for 72 h, and can be employed as a suitable carrier for the controlled delivery of KETM.

### 3.11. Kinetic Modeling

Zero order, first order, Higuchi, and Korsmeyer–Peppas models are applied to release data in order to analyze the release pattern of the drug from the fabricated hydrogels. It is indicated from Table 3 that all formulations follow the first order release kinetics because the “r^2^” values of first order are near 1. Hence, the release rate of the drug from the developed hydrogels is dependent on the concentration of the drug encapsulated inside their networks. The drug release mechanism is determined by the Korsmeyer–Peppas equation. “n” values for most of the formulations are greater than 0.5 indicating the non-Fickian diffusion [62,63], which indicates that the KETM release from hydrogel was at least partially controlled by viscoelastic relaxation of polymer during medium penetration.

### 3.12. In-Vivo Study

**Figure 8:**
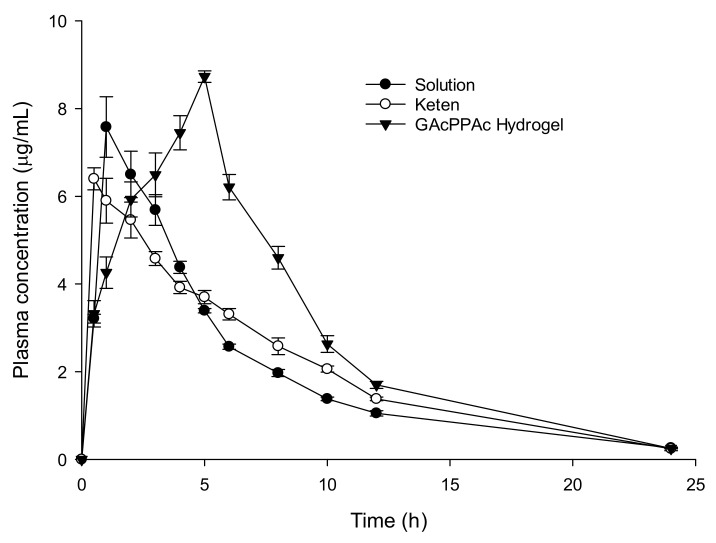
Plasma concentration vs. time plot of KETM administered as the oral solution, keten, and GAcPAA hydrogels to healthy rabbits. (*n* = 3).

An in-vivo study was carried out on nine healthy rabbits to evaluate the effect of GAcPAAc hydrogel on the oral availability of the drug. The plasma drug concentration vs. time is indicated in Figure 8, for drug solution, keten, and KETM-loaded GAcPAAc hydrogels. We can see that plasma concentration of drug solution and keten is less than the plasma concentration of loaded GAcPAAc hydrogels. The drug release is in the pattern of loaded GAcPAAc hydrogels > keten > drug solution. We can conclude that drug release is sustained highly by loaded GAcPAAc hydrogels for a long period of time compared to drug solution and keten. A significant difference is seen in pharmacokinetics parameter of these three groups. In a recent study, the low T_max_ values of drug solution (1.00 ± 0.00) and keten (0.50 ± 0.00) indicated the rapid absorption of the drug compared to loaded GAcPAAc hydrogels (5.00 ± 0.00). The absorption of the drug is much greater with significant differences from GAcPAAc hydrogels revealing the slow drug absorption from the developed hydrogels and, hence, indicating sustained release behavior. Maximum plasma concentration (8.73 ± 0.13 μg/mL) is achieved by loaded GAcPAAc hydrogels compared to the drug solution (7.58 ± 115.67 μg/mL) and keten (6.42 ± 0.25 μg/mL) after 24 h. Hence, the C_max_ values of loaded GAcPAAc hydrogels > keten > pure drug, even containing the same concentration of the drug. These all mean that plasma concentration is maintained for a long period of time by the fabricated hydrogels compared to the drug solution and keten. Likewise, a great difference is seen in other pharmacokinetic parameters of the drug solution, keten, and loaded GAcPAAc hydrogels, respectively. AUC_0–∞_ (μg/mL*h) value of fabricated hydrogels (72.35 ± 2.31) is significantly greater than that of the drug solution (49.16 ± 2.36) and keten (52.47 ± 0.57) (*p* < 0.05). Conclusively, the above results indicate that bioavailability of KETM is enhanced significantly for a long period of time by fabricated hydrogels compared to the drug solution and the commercial product (keten). Hence, the developed hydrogels could be employed further for clinical purposes in the future.

## 4. Conclusions

In the current study, pH-responsive glutamic acid based hydrogels were developed successfully by the free radical polymerization technique for controlled release of Ketorolac tromethamine. FTIR presented the formation of a stable network of polymeric hydrogels. High thermal stability, loss in crystallinity of the polymer, and smooth porous surface of the developed hydrogels was evaluated by TGA, DSC, PXRD, and SEM respectively. pH sensitive nature of the fabricated hydrogels was revealed by the swelling and drug release studies. Dynamic swelling, drug loading, and a percent of the drug released increased with the increasing concentration of glutamic acid and acrylic acid, and decreased with the increasing concentration of EGDMA. Similarly, an increase in percent porosity was seen with the increase in the glutamic acid and acrylic acid concentrations while inverse behavior was perceived with EGDMA concentration. An in-vivo study indicated that developed hydrogels exhibited a significant release and absorption of a drug with high C_max_ and T_max_ values compared to a pure drug solution and keten. Based on the above results, we can conclude that the developed hydrogels could be investigated further for the controlled delivery of other hydrophilic drugs and NSAIDs.

## Figures and Tables

**Table 1:** Feed ratio scheme for formulations, gel fraction, and polymer volume fraction of GAcPAAc hydrogels.

F. Code	GA g/100 g	AAc g/100 g	EGDMA g/100 g	Gel Fraction (%)	Polymer Volume Fraction	
					pH 1.2	pH 7.4
GAF-1	0.50	25	1.0	91.62	0.298	0.107
GAF-2	1.00	25	1.0	93.23	0.294	0.104
GAF-3	1.50	25	1.0	95.01	0.287	0.102
GAF-4	0.50	20	1.0	90.84	0.310	0.113
GAF-5	0.50	25	1.0	91.62	0.298	0.107
GAF-6	0.50	30	1.0	92.90	0.260	0.102
GAF-7	0.50	25	1.0	91.62	0.298	0.107
GAF-8	0.50	25	1.5	93.16	0.304	0.115
GAF-9	0.50	25	2.0	94.07	0.330	0.124

A fixed quantity of 0.5 g of ammonium persulfate (APS) was used for all formulations.

**Table 2:** Drug loading of GAcPAAc hydrogels.

Formulation Code	Amount of DS Loaded in Hydrogels (mg)/400 mg of Dry Gels	
	(Extraction Method)	(Weight Method)
GAF-1	147.90 ± 0.62	146.21 ± 0.98
GAF-2	156.02 ± 0.71	154.18 ± 0.83
GAF-3	160.20 ± 0.96	161.25 ± 1.20
GAF-4	097.89 ± 1.23	096.19 ± 1.04
GAF-5	147.90 ± 0.62	146.21 ± 0.98
GAF-6	155.90 ± 1.13	156.43 ± 1.08
GAF-7	147.90 ± 0.62	146.21 ± 0.98
GAF-8	113.83 ± 0.88	115.29 ± 1.24
GAF-9	092.03 ± 1.02	090.49 ± 1.01

**Table 3:** Kinetic modeling release of KETM from GAcPAAc hydrogels.

F. Code	Zero Order r^2^	First Order r^2^	Higuchi r^2^	Korsmeyer-Peppas	
				r^2^	n
GAF-1	0.8994	0.9715	0.9029	0.9496	0.5972
GAF-2	0.9043	0.9832	0.9813	0.9637	0.5267
GAF-3	0.8408	0.9914	0.9489	0.9622	0.4595
GAF-4	0.8974	0.9846	0.9781	0.9400	0.7102
GAF-5	0.8994	0.9715	0.9029	0.9496	0.5972
GAF-6	0.8678	0.9827	0.9626	0.9564	0.4859
GAF-7	0.8994	0.9715	0.9029	0.9496	0.5972
GAF-8	0.9217	0.9896	0.9891	0.9461	0.8414
GAF-9	0.9314	0.9895	0.9889	0.9678	0.8515

## Data Availability

Not applicable.

## References

[1] Goerne L., López García M., Rodríguez Grada G., Ortiz Pérez I., Gómez López E., Lemus A. (2013). Obtaining of sol-gel ketorolac-silica nanoparticles: Characterization and drug release kinetics. J. Nanomater..

[2] Puglia C., Filosa R., Peduto A., De Caprariis P., Rizza L., Bonina F., Blasi P. (2006). Evaluation of alternative strategies to optimize ketorolac transdermal delivery. AAPS Pharmscitech.

[3] Wagh P., Mujumdar A., Naik J.B. (2019). Preparation and characterization of ketorolac tromethamine-loaded ethyl cellulose micro-/nanospheres using different techniques. Part. Sci. Technol..

[4] Alsarra I.A., Bosela A., Ahmed S., Mahrous G. (2005). Proniosomes as a drug carrier for transdermal delivery of ketorolac. Eur. J. Pharm. Biopharm..

[5] Mathew S.T., Devi S.G., Sandhya K. (2007). Formulation and evaluation of ketorolac tromethamine-loaded albumin microspheres for potential intramuscular administration. AAPS Pharmscitech.

[6] Mamidi N., Delgadillo R.M.V. (2021). Design, fabrication and drug release potential of dual stimuli-responsive composite hydrogel nanoparticle interfaces. Colloids Surf. B Biointerfaces.

[7] Mamidi N., Velasco Delgadillo R.M., Barrera E.V. (2021). Covalently Functionalized Carbon Nano-Onions Integrated Gelatin Methacryloyl Nanocomposite Hydrogel Containing γ-Cyclodextrin as Drug Carrier for High-Performance pH-Triggered Drug Release. Pharmaceuticals.

[8] Suhail M., Rosenholm J.M., Minhas M.U., Badshah S.F., Naeem A., Khan K.U., Fahad M. (2019). Nanogels as drug-delivery systems: A comprehensive overview. Ther. Deliv..

[9] Mamidi N., Castrejón J.V., González-Ortiz A. (2020). Rational design and engineering of carbon nano-onions reinforced natural protein nanocomposite hydrogels for biomedical applications. J. Mech. Behav. Biomed. Mater..

[10] Mamidi N., Zuníga A.E., Villela-Castrejón J. (2020). Engineering and evaluation of forcespun functionalized carbon nano-onions reinforced poly (ε-caprolactone) composite nanofibers for pH-responsive drug release. Mater. Sci. Eng. C.

[11] Al-Tabakha M.M., Khan S.A., Ashames A., Ullah H., Ullah K., Murtaza G., Hassan N. (2021). Synthesis, Characterization and Safety Evaluation of Sericin-Based Hydrogels for Controlled Delivery of Acyclovir. Pharmaceuticals.

[12] Shih I.-L., Van Y.-T., Shen M.-H. (2004). Biomedical applications of chemically and microbiologically synthesized poly (glutamic acid) and poly (lysine). Mini Rev. Med. Chem..

[13] Hopkins T.E., Pawlow J.H., Koren D.L., Deters K.S., Solivan S.M., Davis J.A., Gómez F.J., Wagener K.B. (2001). Chiral polyolefins bearing amino acids. Macromolecules.

[14] Suhail M., Wu P.-C., Minhas M.U. (2020). Using carbomer-based hydrogels for control the release rate of diclofenac sodium: Preparation and in vitro evaluation. Pharmaceuticals.

[15] Zahra Q., Minhas M.U., Khan S., Wu P.-C., Suhail M., Iqbal R., Bashir M. (2021). Fabrication of polyethylene glycol hydrogels with enhanced swelling; loading capacity and release kinetics. Polym. Bull..

[16] Suhail M., Khan A., Rosenholm J.M., Minhas M.U., Wu P.-C. (2021). Fabrication and characterization of diclofenac sodium loaded hydrogels of sodium alginate as sustained release carrier. Gels.

[17] Suhail M., Wu P.-C., Minhas M.U. (2021). Development and characterization of pH-sensitive chondroitin sulfate-co-poly (acrylic acid) hydrogels for controlled release of diclofenac sodium. J. Saudi Chem. Soc..

[18] Zia M.A., Sohail M., Minhas M.U., Sarfraz R.M., Khan S., de Matas M., Hussain Z., Abbasi M., Shah S.A., Kousar M. (2020). HEMA based pH-sensitive semi IPN microgels for oral delivery; a rationale approach for ketoprofen. Drug Dev. Ind. Pharm..

[19] Khalid I., Ahmad M., Minhas M.U., Barkat K. (2018). Preparation and characterization of alginate-PVA-based semi-IPN: Controlled release pH-responsive composites. Polym. Bull..

[20] Badshah S.F., Akhtar N., Minhas M.U., Khan K.U., Khan S., Abdullah O., Naeem A. (2021). Porous and highly responsive cross-linked β-cyclodextrin based nanomatrices for improvement in drug dissolution and absorption. Life Sci..

[21] Sarfraz R., Khan H., Mahmood A., Ahmad M., Maheen S., Sher M. (2015). Formulation and evaluation of mouth disintegrating tablets of atenolol and atorvastatin. Indian J. Pharm. Sci..

[22] Khan K.U., Minhas M.U., Sohail M., Badshah S.F., Abdullah O., Khan S., Munir A., Suhail M. (2021). Synthesis of PEG-4000-co-poly (AMPS) nanogels by cross-linking polymerization as highly responsive networks for enhancement in meloxicam solubility. Drug Dev. Ind. Pharm..

[23] Suhail M., Hsieh Y.-H., Khan A., Minhas M.U., Wu P.-C. (2021). Preparation and In Vitro Evaluation of Aspartic/Alginic Acid Based Semi-Interpenetrating Network Hydrogels for Controlled Release of Ibuprofen. Gels.

[24] Bukhari S.M.H., Khan S., Rehanullah M., Ranjha N.M. (2015). Synthesis and characterization of chemically cross-linked acrylic acid/gelatin hydrogels: Effect of pH and composition on swelling and drug release. Int. J. Polym. Sci..

[25] Nasir N., Ahmad M., Minhas M.U., Barkat K., Khalid M.F. (2019). pH-responsive smart gels of block copolymer [pluronic F127-co-poly (acrylic acid)] for controlled delivery of Ivabradine hydrochloride: Its toxicological evaluation. J. Polym. Res..

[26] Peppas N.A., Sahlin J.J. (1989). A simple equation for the description of solute release. III. Coupling of diffusion and relaxation. Int. J. Pharm..

[27] Sohail M., Ahmad M., Minhas M.U., Ali L., Khalid I., Rashid H. (2015). Controlled delivery of valsartan by cross-linked polymeric matrices: Synthesis, in vitro and in vivo evaluation. Int. J. Pharm..

[28] Sethi P. (2008). Quantitative Analysis of Drugs in Pharmaceutical Formulations.

[29] Dergunov S.A., Nam I.K., Mun G.A., Nurkeeva Z.S., Shaikhutdinov E.M. (2005). Radiation synthesis and characterization of stimuli-sensitive chitosan–polyvinyl pyrrolidone hydrogels. Radiat. Phys. Chem..

[30] Harish N., Prabhu P., Charyulu R., Gulzar M., Subrahmanyam E. (2009). Formulation and evaluation of in situ gels containing clotrimazole for oral candidiasis. Indian J. Pharm. Sci..

[31] Hussain T., Ranjha N.M., Shahzad Y. (2011). Swelling and controlled release of tramadol hydrochloride from a pH-sensitive hydrogel. Des. Monomers Polym..

[32] Khalid I., Ahmad M., Minhas M.U., Barkat K. (2018). Synthesis and evaluation of chondroitin sulfate based hydrogels of loxoprofen with adjustable properties as controlled release carriers. Carbohydr. Polym..

[33] Gao Q., Zhang C., Wang M., Wu Y., Gao C., Zhu P. (2020). Injectable pH-responsive poly (γ-glutamic acid)-silica hybrid hydrogels with high mechanical strength, conductivity and cytocompatibility for biomedical applications. Polymer.

[34] Panda P.K., Yang J.-M., Chang Y.-H., Su W.-W. (2019). Modification of different molecular weights of chitosan by p-Coumaric acid: Preparation, characterization and effect of molecular weight on its water solubility and antioxidant property. Int. J. Biol. Macromol..

[35] Moharram M., Khafagi M. (2007). Application of FTIR spectroscopy for structural characterization of ternary poly (acrylic acid)–metal–poly (vinyl pyrrolidone) complexes. J. Appl. Polym. Sci..

[36] Begum M.Y., Shaik M.R., Abbulu K., Sudhakar M. (2012). Ketorolac tromethamine loaded liposomes of long alkyl chain lipids: Development, characterization and in vitro performance. Int. J. PharmTech Res..

[37] Aşik M.D., Uğurlu N., Yülek F., Tuncer S., Mustafa T., Denkbaş E.B. (2013). Ketorolac tromethamine loaded chitosan nanoparticles as a nanotherapeutic system for ocular diseases. Hacet. J. Biol. Chem..

[38] Waghulde M., Mujumdar A., Naik J. (2019). Preparation and characterization of miglitol-loaded Poly (d, l-lactide-co-glycolide) microparticles using high pressure homogenization-solvent evaporation method. Int. J. Polym. Mater. Polym. Biomater..

[39] Khalid I., Ahmad M., Usman Minhas M., Barkat K., Sohail M. (2018). Cross-Linked Sodium Alginate-g-Poly (Acrylic Acid) Structure: A Potential Hydrogel Network for Controlled Delivery of Loxoprofen Sodium. Adv. Polym. Technol..

[40] Manocha B., Margaritis A. (2010). A novel Method for the selective recovery and purification of γ-polyglutamic acid from Bacillus licheniformis fermentation broth. Biotechnol. Prog..

[41] Khachatoorian R., Petrisor I.G., Yen T.F. (2004). Prediction of plugging effect of biopolymers using their glass transition temperatures. J. Pet. Sci. Eng..

[42] Suhail M., Fang C.-W., Khan A., Minhas M.U., Wu P.-C. (2021). Fabrication and In Vitro Evaluation of pH-Sensitive Polymeric Hydrogels as Controlled Release Carriers. Gels.

[43] Sarika P., James N.R., Raj D.K. (2016). Preparation, characterization and biological evaluation of curcumin loaded alginate aldehyde–gelatin nanogels. Mater. Sci. Eng. C.

[44] Sohail M., Ahmad M., Minhas M.U., Ali L., Munir A., Khalid I. (2014). Synthesis and characterization of graft PVA composites for controlled delivery of valsartan. Lat. Am. J. Pharm..

[45] Liu C., Chen Y., Chen J. (2010). Synthesis and characteristics of pH-sensitive semi-interpenetrating polymer network hydrogels based on konjac glucomannan and poly (aspartic acid) for in vitro drug delivery. Carbohydr. Polym..

[46] Krisch E., Gyarmati B., Barczikai D., Lapeyre V., Szilágyi B.Á., Ravaine V., Szilagyi A. (2018). Poly (aspartic acid) hydrogels showing reversible volume change upon redox stimulus. Eur. Polym. J..

[47] Gyenes T., Torma V., Gyarmati B., Zrínyi M. (2008). Synthesis and swelling properties of novel pH-sensitive poly (aspartic acid) gels. Acta Biomater..

[48] Çaykara T., Turan E. (2006). Effect of the amount and type of the crosslinker on the swelling behavior of temperature-sensitive poly (N-tert-butylacrylamide-co-acrylamide) hydrogels. Colloid Polym. Sci..

[49] Teijón C., Olmo R., Blanco M.D., Teijón J.M., Romero A. (2006). Effect of the crosslinking degree and the nickel salt load on the thermal decomposition of poly (2-hydroxyethyl methacrylate) hydrogels and on the metal release from them. J. Colloid Interface Sci..

[50] Teijon J., Trigo R., Garcia O., Blanco M. (1997). Cytarabine trapping in poly (2-hydroxyethyl methacrylate) hydrogels: Drug delivery studies. Biomaterials.

[51] Vazquez B., Gurruchaga M., Goni I. (1995). Hydrogels based on graft copolymerization of HEMA/BMA mixtures onto soluble gelatin: Swelling behaviour. Polymer.

[52] Khanum H., Ullah K., Murtaza G., Khan S.A. (2018). Fabrication and in vitro characterization of HPMC-g-poly (AMPS) hydrogels loaded with loxoprofen sodium. Int. J. Biol. Macromol..

[53] Lee C.-T., Huang C.-P., Lee Y.-D. (2007). Synthesis and characterizations of amphiphilic poly (l-lactide)-grafted chondroitin sulfate copolymer and its application as drug carrier. Biomol. Eng..

[54] Murthy P.K., Mohan Y.M., Sreeramulu J., Raju K.M. (2006). Semi-IPNs of starch and poly (acrylamide-co-sodium methacrylate): Preparation, swelling and diffusion characteristics evaluation. React. Funct. Polym..

[55] Rashid H., Ahmad M., Minhas M.U., Sohail M., Aamir M.F. (2015). Synthesis and Characterization of Poly (hydroxyethyl methacrylate-co-methacrylic acid) Cross Linked Polymeric Network for the Delivery of Analgesic Agent. J. Chem. Soc. Pak..

[56] Zhu C., Tang N., Gan J., Zhang X., Li Y., Jia X., Cheng Y. (2021). A pH-sensitive semi-interpenetrating polymer network hydrogels constructed by konjac glucomannan and poly (γ-glutamic acid): Synthesis, characterization and swelling behavior. Int. J. Biol. Macromol..

[57] Ullah K., Khan S.A., Murtaza G., Sohail M., Manan A., Afzal A. (2019). Gelatin-based hydrogels as potential biomaterials for colonic delivery of oxaliplatin. Int. J. Pharm..

[58] Şanlı O., Ay N., Işıklan N. (2007). Release characteristics of diclofenac sodium from poly (vinyl alcohol)/sodium alginate and poly (vinyl alcohol)-grafted-poly (acrylamide)/sodium alginate blend beads. Eur. J. Pharm. Biopharm..

[59] Akhtar M.F., Ranjha N.M., Hanif M. (2015). Effect of ethylene glycol dimethacrylate on swelling and on metformin hydrochloride release behavior of chemically crosslinked pH–sensitive acrylic acid–polyvinyl alcohol hydrogel. DARU J. Pharm. Sci..

[60] Rokhade A.P., Agnihotri S.A., Patil S.A., Mallikarjuna N.N., Kulkarni P.V., Aminabhavi T.M. (2006). Semi-interpenetrating polymer network microspheres of gelatin and sodium carboxymethyl cellulose for controlled release of ketorolac tromethamine. Carbohydr. Polym..

[61] Dasankoppa F.S., Ningangowdar M., Sholapur H. (2012). Formulation and evaluation of controlled porosity osmotic pump for oral delivery of ketorolac. J. Basic Clin. Pharm..

[62] Maziad N.A., El-Hamouly S., Zied E., El Kelani T.A., Nasef N.R. (2015). Radiation preparation of smart hydrogel has antimicrobial properties for controlled release of ciprofloxacin in drug delivery systems. Asian J. Pharm. Clin. Res..

[63] Shoaib M.H., Tazeen J., Merchant H.A., Yousuf R.I. (2006). Evaluation of drug release kinetics from ibuprofen matrix tablets using HPMC. Pak. J. Pharm. Sci..

